# Few-Shot Fault Diagnosis Based on an Attention-Weighted Relation Network

**DOI:** 10.3390/e26010022

**Published:** 2023-12-24

**Authors:** Li Xue, Aipeng Jiang, Xiaoqing Zheng, Yanying Qi, Lingyu He, Yan Wang

**Affiliations:** 1 HDU-ITMO Joint Institute, Hangzhou Dianzi University, Hangzhou 310018, China; 212320053@hdu.edu.cn; 2 School of Automation, Hangzhou Dianzi University, Hangzhou 310018, China; zhengxiaoqing@hdu.edu.cn (X.Z.); qyy012827@hdu.edu.cn (Y.Q.); 232320071@hdu.edu.cn (L.H.); wangyan0930@hdu.edu.cn (Y.W.)

**Keywords:** fault diagnosis, energy conversion systems, relation network, attention mechanism, pneumatic control valve

## Abstract

As energy conversion systems continue to grow in complexity, pneumatic control valves may exhibit unexpected anomalies or trigger system shutdowns, leading to a decrease in system reliability. Consequently, the analysis of time-domain signals and the utilization of artificial intelligence, including deep learning methods, have emerged as pivotal approaches for addressing these challenges. Although deep learning is widely used for pneumatic valve fault diagnosis, the success of most deep learning methods depends on a large amount of labeled training data, which is often difficult to obtain. To address this problem, a novel fault diagnosis method based on the attention-weighted relation network (AWRN) is proposed to achieve fault detection and classification with small sample data. In the proposed method, fault diagnosis is performed through the relation network in few-shot learning, and in order to enhance the representativeness of feature extraction, the attention-weighted mechanism is introduced into the relation network. Finally, in order to verify the effectiveness of the method, a DA valve fault dataset is constructed, and experimental validation is performed on this dataset and another benchmark PU rolling bearing fault dataset. The results show that the accuracy of the network on DA is 99.15%, and the average accuracy on PU is 98.37%. Compared with the state-of-the-art diagnosis methods, the proposed method achieves higher accuracy while significantly reducing the amount of training data.

## 1. Introduction

In the control of production processes, pneumatic control valves serve as energy conversion devices for regulating various process parameters such as medium flow, pressure, temperature, and liquid level. As an energy conversion device, a pneumatic control valve may experience unpredictable operational abnormalities potentially diminishing the system’s reliability. Accurately and promptly identifying malfunctions in pneumatic valves during operation is crucial for ensuring their safe operation, avoiding economic losses, and preventing catastrophic accidents. As such, fault diagnosis of pneumatic valve equipment is an essential component of intelligent manufacturing. It helps to maintain the safety and health of mechanical equipment throughout its service life [1].

In recent years, deep learning-based fault diagnosis has made significant progress due to the rapid development of deep learning. Unlike traditional methods that rely on expert experience and manual feature extraction operations, which can be time-consuming, error-prone, and inaccurate, deep learning-based methods enable accurate and efficient fault diagnosis in an end-to-end manner [2]. In 2006, Bartys et al. [3] proposed the DAMATICS valve fault diagnosis model, which benchmarked a total of 19 fault tests present in four main functional blocks, providing a benchmark for subsequent valve fault diagnosis studies. In the same year, Witczak et al. [4] first proposed the use of neural networks to diagnose valve faults and applied the DAMATICS model to generate fault data to complete classification and detection. In 2016, Cabeza et al. [5] proposed the use of Hopfield neural networks to solve data loss and information scarcity problems in data acquisition systems. In 2017, Oliveira et al. [6] applied weightless neural networks for the detection and diagnosis of dynamic systems, which use neurons based on RAM devices and can adjust parameters more easily and quickly. In the same year, José et al. [7] used artificial neural networks as a complement to conventional detectors through parameter identification, correcting errors in the thresholds, and allowing the detectors to demonstrate better fault detection performance. In 2021, Andrade et al. [8] proposed the use of a non-linear autoregressive neural network model with exogenous inputs to generate residuals and applied isolation and decision tree methods to diagnose pneumatic regulating valve faults through residuals. In 2022, Garg et al. [9] proposed the use of unsupervised and semi-supervised deep learning methods for anomaly detection and judgment of valve data. Overall, these studies demonstrate the significant potential of deep learning-based methods for improving the accuracy and efficiency of fault diagnosis in pneumatic control valves. They have contributed to the development of new and innovative fault diagnosis approaches, and are expected to have a profound impact on the field of intelligent manufacturing.

However, these methods require large amounts of labeled training data and expensive computational resources, limiting their potential for practical application [10]. In addition, collecting sufficient labeled fault data in special environments and fault states is challenging and labor-intensive, making it difficult to obtain the necessary data for effective fault diagnosis. To the best of our knowledge, there is no previous research on accurately diagnosing pneumatic valve faults with limited sample data. To address this issue, this paper proposes a few-shot valve fault detection method based on AWRN. The proposed method uses an embedding layer to extract sample information and trains a metric convolution network to map similar samples closer together in space and dissimilar samples farther apart, thereby achieving more accurate classification. Furthermore, an attention mechanism is introduced in a weighted manner after the embedding layer to improve the feature extraction ability and classification accuracy with small samples. This approach is a promising solution to the problem of accurate fault diagnosis with limited sample data. Overall, this paper provides valuable insights into the development of few-shot learning methods for pneumatic valve fault diagnosis, which has been an underexplored area in the field. By addressing the limitations of current methods and proposing a novel approach, this study contributes to the advancement of intelligent manufacturing and the maintenance of safe and healthy mechanical equipment.

The main contributions of this paper can be summarized as follows:

1. To address the problem of accurately diagnosing pneumatic valve faults with limited sample data and to enhance the representative capability of feature extraction, this paper proposes a weighted attention relation network (AWRN) that introduces the attention mechanism to the relation network in a weighted manner. The applicability of this method extends beyond pneumatic valve failures and can be extrapolated to other industrial systems.
2. To alleviate the lack of a publicly available valve fault dataset, we constructed a benchmark valve fault dataset based on the DAMATICS model, and make it publicly available at https://github.com/Levin727/DA$_$database.git (accessed on 1 November 2023).
3. To increase the reliability of the system, we meticulously scrutinize and draw a detailed comparison between two distinct attention-weighted methods. Additionally, we rigorously derive the hyperparameters that are relevant to the network model. The experimental results demonstrate that the proposed network achieves high accuracy even with significantly reduced amounts of training data, and has a strong generalization capability to different tasks.

The rest of the paper is organized as follows: Section 2 reviews the related work. Section 3 describes the classification method and fault diagnosis process of the proposed model. Section 4 constructs the DA dataset and presents the PU public dataset. Section 5 shows the experimental results. Section 6 summarizes the full paper.

## 2. Related Work

When faced with limited labeled samples, a popular approach for intelligent mechanical fault diagnosis is the few-shot learning method. Zhang et al. [11] were the first to use this approach in fault diagnosis in 2019, and since then, many studies have used few-shot learning methods [12,13,14,15] to extract input signal features. Few-shot learning involves training a model with only a small amount of sample data, and the model must learn general patterns or features from these limited samples to handle a wider range of data [16]. Few-shot learning methods can be categorized into two main types: transfer-based learning methods and meta-learning-based methods. Transfer-based learning methods transfer existing knowledge to a new task, typically by using existing models to initialize new models, which speeds up training and improves model performance. Representative algorithms include MAML [17]. Meta-learning-based methods involve training with a large number of similar tasks, allowing the model to adapt more quickly to a new task. Chen et al. [18] provide a valuable overview of the development of deep transfer learning-based bearing fault diagnosis since 2016, offering valuable guidance and important insights for the current study. Few-shot learning methods based on meta-learning fall into four main categories: metric-based methods, model-based methods, optimization-based methods, and data augmentation-based methods. This paper focuses on metric-based methods for few-shot learning. The operational symbols are shown in Table 1.

Few-shot learning based on metric methods aims to classify samples by measuring their similarity. To achieve this, metric networks first extract sample information using an embedding layer and train a metric function that maps similar samples to a closer space and dissimilar samples to a more distant space. Siamese networks [19], prototypical networks [20], and relation networks [21] are some of the classical methods used for this purpose. Siamese networks rely on feature vectors obtained from neural networks and use Euclidean distances to calculate similarity. Recently, many scholars [22,23,24] have also proposed new models based on Siamese networks for fault diagnosis applications. Prototypical networks create a prototype representation for each classification based on a limited number of labeled samples, and the distance between the prototype vector and the query point of the classification is used to determine classification. Many recent studies [25,26,27] have used Prototypical networks to learn feature mappings for fault diagnosis with limited samples. For example, Chen et al. [28] proposed MoProNet for addressing cross-domain few-shot rotating machinery fault diagnosis. MoProNet employs a progressive update strategy for the support encoder to resolve prototype oscillation issues, thereby enhancing network performance. Relation networks (RNs) obtain feature vectors through multiple convolution layers and analyze the degree of matching by building a neural network to calculate the distance between two samples.

**Figure 1:**
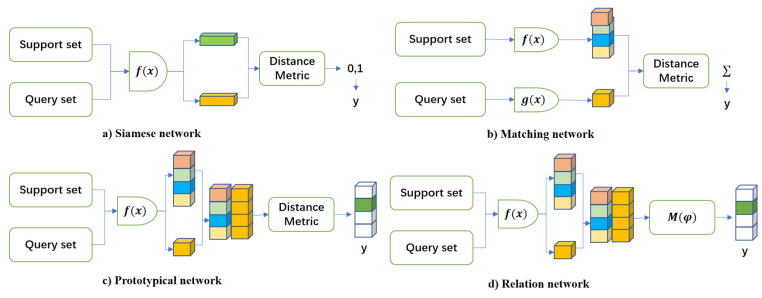
Metric-based few-shot learning network.

As illustrated in Figure 1, compared to Siamese networks and prototypical networks, relation networks can provide a non-linear classifier $M\left(\varphi \right)$ that can learn relationships more accurately. Dynamic evaluation functions are better than static evaluation functions in time series anomaly detection, and a non-linear representation can evaluate relationships more accurately, making relation networks more effective for fault signal analysis [9]. Therefore, in this study, relation networks were selected for classifying and analyzing the fault dataset. The proposed attention-weighted relation network in this paper builds upon the foundation of the relation networks. Through the incorporation of an attention-weighted method, the overall system performance is enhanced, leading to improved classification effectiveness. The common definitions in relation networks are shown in Table 2.

The core concept of relation networks is to use the relationship between the support set and the query set for classification in few-shot learning tasks. This process consists of two stages: first, the embedding module extracts signal features from the support and query sets respectively. Then, a similarity measure network is constructed to classify the signal features by comparing the similarity between the support and query set features. The optimization formula is represented by the following Equation [21]:

(1) $$\theta_{F}^{*},\theta_{M}^{*}\leftarrow \underset{\theta_{F},\theta_{M}}{argmin\;}L_{MSE}\left(\theta_{F},\theta_{M}\right)$$

Here, $\theta_{F}$ and $\theta_{M}$ are the weights of the embedding layer and the similarity measure network, respectively. The embedding layers of the support and query sets share the same weights. The optimal parameters are obtained through iterative optimization.

In 2020, Chang et al. [29] introduced relation networks to fault diagnosis by proposing a few-shot relation network with 2D data processed by STFT as input and using an attention mechanism to enhance feature extraction. Wu et al. [30] compared meta-learning relation networks (MRNs) with other common transfer methods to evaluate the performance of metric-based networks in meta-learning transfer methods. However, few-shot learning in fault diagnosis is still a relatively unexplored area, and relation networks may misclassify relatively similar signals due to insufficient feature extraction and poor feature representation. Additionally, the network may focus solely on feature extraction without exploiting the contrast between the support and query set features, which is a critical feature of metric-based networks such as relation networks.

To address the issue of insufficient feature extraction capability, several studies have combined transfer learning with the relation network [31,32,33]. This approach involves training a good feature extractor using a large amount of data and then transferring it to the target domain for feature extraction. Additionally, attention mechanisms have been introduced to enhance the representativeness of the constructed model and improve subsequent non-linear classification and classification accuracy. For instance, Yu et al. [34] incorporated an attention mechanism in the feature extraction module to increase the weight of important parts. Chen et al. [35] introduced a spatial attention mechanism to improve the representativeness of the relation network. Zhang et al. [15] proposed the use of self-attention mechanisms in relation networks for few-shot learning to model cross-regional features. Furthermore, Gkanatsios et al. [36] proposed multi-headed attention mechanism relation networks to capture the properties of datasets of different sizes while addressing the problem of background category bias in multitasking. Attentional mechanisms mimic human perceptual systems by selectively focusing on salient parts and recording those features, which enhances the accuracy and generalizability of the network.

There are two primary approaches to utilizing attentional networks in the aforementioned studies: one is to incorporate an attentional mechanism in the feature extraction module, and the other is to introduce an attentional mechanism after the feature module. While both approaches enable the network to obtain better features and improve their representativeness, they do not enhance the feature comparison between the support set and the query set, which is required in metric networks. Therefore, in this paper, a special attention-weighted structure is utilized to improve the contrast between the support set and the query set by using the attention mechanism as weights and weighting it onto the support set and the query set, respectively, to obtain better classification results.

## 3. The Proposed AWRN Fault Diagnosis Approach

As an energy conversion device, the control mode of a pneumatic control valve involves the transmission of a 4–20 mA analog signal through the control system. This electric signal is subsequently transformed into a pneumatic signal by the electrical conversion unit within the valve positioner. The pneumatic signal then enters the pneumatic thin-film actuator, where it is converted into kinetic energy, ultimately facilitating the movement of the valve lever.

To address the challenge of high-accuracy diagnosis for pneumatic valve faults with small sample data and to enhance the representative capability of feature extraction, we propose a weighted relation network incorporating an attention strategy, named the attention-weighted relation network (AWRN). The AWRN approach includes a feature extraction module, a parallel attention-weighted module, and a similarity contrast module. Given the support set samples $x_{i}^{S}$ and query set samples $x_{j}^{Q}$ as input, the feature extraction module extracts the support features and query features, which are then combined as $f^{S}$ and $f^{Q}$ and used as input to the parallel attention-weighted module. The parallel attention-weighted module uses a parallel attention mechanism to extract salient features from the faulty data, which are then weighted onto both the support set and the query set features to obtain new support set features $f^{S^{\prime }}$ and new query set features $f^{Q^{\prime }}$ . Finally, the similarity contrast module evaluates the features and outputs the evaluation score.

The benefits of our approach are twofold: First, the parallel attention mechanism enables the extraction of salient features from the sample data that can be better used for classification. Second, the weighting operation on both sets simultaneously can enhance the contrast between the support and query sets, making features of the same class more similar and features of different classes more dissimilar, thus improving the accuracy of the network.

### 3.1. The Proposed AWRN Network

**Figure 2:**
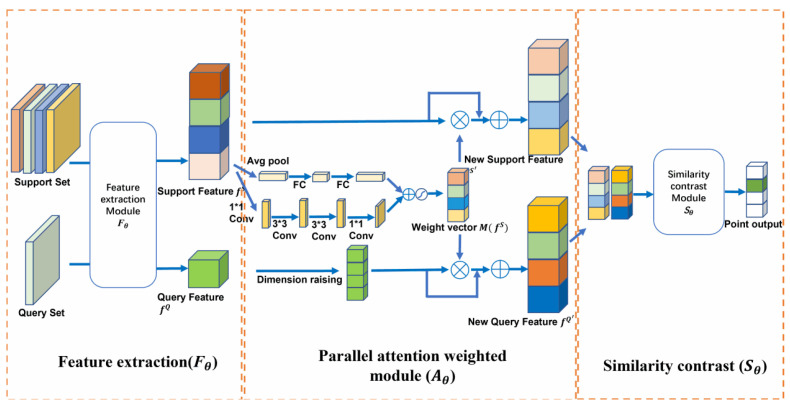
Network architecture of AWRN. For ease of illustration, a four-way and one-shot classification task was chosen for this figure. AWRN consists of three modules, a feature extraction module, a parallel attention-weighted module, and a similarity contrast module. The leftmost rectangle (support/query set) is the scalar and the square represents the 3D tensor. The white boxes indicate the modules and denote the features generated by the support set and query set after the feature extraction module, respectively; they represent the new features obtained after the attention-weighted module. $M(f^{S})$ denotes the attention weight parameter. The output score represents the score obtained after the similarity contrast network, where a value closer to 1 indicates a darker color.

As illustrated in Figure 2, AWRN consists of three modules, a feature extraction module, a parallel attention-weighted module, and a similarity comparison module.

#### 3.1.1. Feature Extraction Module

The feature extraction module is composed of four convolution blocks, each containing 64 channels, and a 3 × 3 convolution kernel. The first two blocks have a 2 × 2 maximum pooling operation and are followed by batch normalization and the ReLU activation function. The convolution block operation can be expressed as follows:

(2) $$\;C_{3\times 3}\left(x^{n}\right)=f\left(w^{n}x^{n}+b^{n}\right)$$

Here, $C_{3\times 3}$ represents a 3 × 3 convolution operation, $x^{n}$ is the input, and $w^{n}$ and $b^{n}$ are the weight and bias of the nth layer of the convolution block, respectively. The maximum pooling layer operation can be expressed as follows:

(3) $$\;P_{n}\left(x^{n}\right)=Max_{2\times 2}\left(x^{n}\right)$$

Here, $Max_{2\times 2}$ represents the maximum pooling operation, and 2 × 2 is the size of the pooling window. The feature extraction module takes support samples $x_{i}^{S}$ and query samples $x_{j}^{Q}$ as inputs and outputs the corresponding features, $F\left(x_{i}^{S}\right)\in R^{1\times H\times W}$ and $F\left(x_{j}^{Q}\right)\in R^{1\times H\times W}$ . For multiple mappings, the support features $F\left(x_{i}^{S}\right)$ are separately processed to form the combined feature $f^{s}\in R^{k\times H\times W}$ and the formula is as follows:

(4) $$\;f^{s}=D\left(F_{\theta }\left(x_{i}^{S}\right)\right),i=1,2,\ldots ,k$$

Here, D represents the concatenation of feature maps at depth, *k* represents the number of categories, and *i* represents each of these categories. The query feature size is $R^{j1HW}$ , where *j* denotes the number of simultaneous branches per iteration. Here, we consider the case where *j* = 1 for convenience. Then, the query feature at this point is $f^{Q}\in R^{1\times H\times W}$ , which is then fed into the attention-weighted module along with the feature map $f_{i}^{S}$ and $f_{j}^{Q}$ , respectively.

When using relation networks for few-shot fault diagnosis, the feature extraction capability may be limited, resulting in less representative features. This can lead to misjudgment when dealing with relatively similar feature vectors. To address this issue, it is necessary to sharpen the subtle differences in the feature vectors and utilize the attention mechanism to enhance the comparison. Then, if an instance is in the same category as the query set, the features will become more similar after attention weighting, while if the instance is not in the same category, the features will generally appear more different, which helps reveal the category distribution more accurately.

#### 3.1.2. Parallel Attention-Weighted Module

To improve the accuracy of the network, we propose adding a parallel attention-weighted network behind the feature network. The utilization of the parallel attention mechanism proves effective in strategically emphasizing dynamic trends within time series data, facilitating the identification of anomalies and mutations. Concurrently, this mechanism enables the model to concentrate on distinct portions of the data across various scales. Specifically, for a given support feature $f^{s}\in R^{k\times H\times W}$ , we use the attention module $M\left(f^{s}\right)\in R^{k\times H\times W}$ to infer a three-dimensional weight vector that is then applied to both the support features $f^{s}$ and the query feature $f^{Q}\in R^{1\times H\times W}$ . Here, we demonstrated that the attention-weighted method, when using the support feature, outperforms the method that relies on the query feature, as evidenced by subsequent experiments. This process results in a special weighting architecture with new weighted support features $f^{S^{\prime }}$ and query features $f^{Q^{\prime }}$ , which are calculated as follows [37]:

(5) $$f^{S^{\prime }}=A_{\theta }\left(f^{S}\right)=f^{S}+f^{S}⊗M\left(f^{S}\right)$$

(6) $$f^{Q^{\prime }}=A_{\theta }\left({f^{Q}}^{k}\right)={f^{Q}}^{k}+{f^{Q}}^{k}⊗M\left(f_{i}^{S}\right)$$

Here, ⊗ denotes element-level multiplication, and ${f^{Q}}^{k}$ denotes dimension raising. To implement the parallel attention mechanism, both channel attention and spatial attention can be used. Channel attention emphasizes attention to channel features in a given input, while spatial attention branches emphasize features at different locations. The attention mechanism is then fused into (5) and (6), and rewritten as follows:

(7) $$f^{S^{\prime }}=f^{S}*\left(1+\sigma \left(BN\left(MLP\left(Avg\left(f_{i}^{S}\right)\right)\right)+BN\left(C_{1\times 1}\left(C_{3\times 3}\left(C_{3\times 3}\left(C_{1\times 1}\left(f_{i}^{S}\right)\right)\right)\right)\right)\right)\right)$$

(8) $$f^{Q^{\prime }}={f^{Q}}^{k}*\left(1+\sigma \left(BN\left(MLP\left(Avg\left(f_{i}^{S}\right)\right)\right)+BN\left(C_{1\times 1}\left(C_{3\times 3}\left(C_{3\times 3}\left(C_{1\times 1}\left(f_{i}^{S}\right)\right)\right)\right)\right)\right)\right)$$

Here, *BN* denotes batch normalization, *MLP* denotes multi-layer perceptron operation, and Avg denotes average pooling operation. The new support features and query features obtained through (7) and (8) are then inputted to the similarity contrast module.

#### 3.1.3. Similarity Contrast Module

The similarity contrast module comprises two convolution layers, each with 64 channels and a kernel size of 3 × 3. After each convolution layer, batch normalization, ReLU activation, and 2 × 2 max pooling are applied, followed by two fully connected layers. The similarity scores are computed using the ReLU activation function and sigmoid function normalization, and the fully connected operation is defined as follows:

(9) $$FC\left(x^{n}\right)=g\left(\alpha^{n}x^{n}+\beta^{n}\right)$$

Here, *FC* represents the fully concatenated operation, $x^{n}$ denotes the input, and $\alpha^{n}$ and $\beta^{n}$ denote the weight and bias of the nth layer of the fully connected layer, respectively. The similarity contrast module $S_{\theta }$ is capable of computing the relationship score between the adjusted support feature $f^{S^{\prime }}$ and query feature $f^{Q^{\prime }}$ . Specifically, the output of $S_{\theta }$ is represented as follows:

(10) $$r_{i,j}=S_{\theta }\left(f^{S^{\prime }},f^{Q^{\prime }}\right)$$

Here, $r_{i,j}$ is the score obtained after a similarity contrast network, which reflects the similarity between the support set and the query set. A higher relationship score implies that the support and query sets belong to the same category, while a lower relationship score indicates that they belong to different categories.

**Figure 3:**
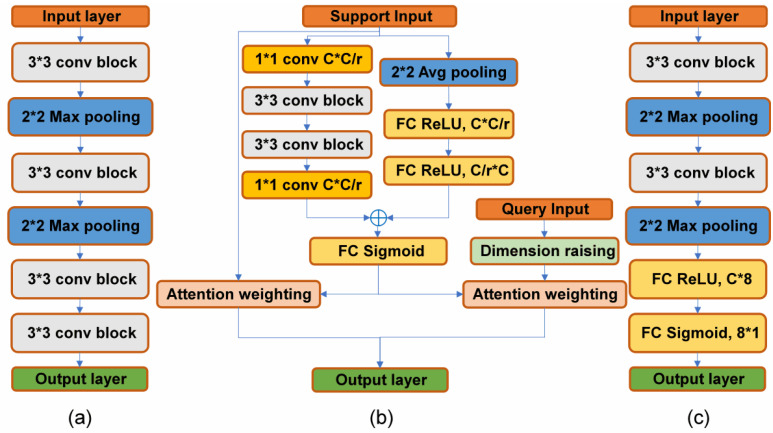
Details of the structure in the AWRN network. (**a**) Feature extraction module; (**b**) parallel attention-weighted module; (**c**) similarity contrast module.

To facilitate comprehension, Figure 3 provides a detailed illustration of the feature extraction module, the parallel attention-weighted module, and the similarity contrast module. The channel size (C) is fixed at 64, and the number of channels of all gray convolution blocks is also set to C. The attenuation ratio (r) is used to increase the coding implication and reduce the computational effort. The term “attention weighted” refers to the process of applying attention weights to the target, as shown in (7) and (8).

### 3.2. The Training Algorithm of AWRN

To explain the overall optimization objective, we can rewrite (10) as follows:

(11) $$r_{i,j}=S_{\theta }\left(A_{\theta }\left(D\left(F_{\theta }\left(x_{i}^{S}\right)\right)\right),A_{\theta }\left(F_{\theta }\left(x_{j}^{Q}\right)\right)\right)i=1,2,\cdots ,k$$

For training the optimal model, we used the mean square error (MSE) to calculate the loss function of the AWRN:

(12) $$L_{MSE}=\sum_{i=1}^{N}\sum_{j=1}^{K}{\left(r_{i,j}-1\cdot \left(y_{i}==y_{j}\right)\right)}^{2}$$

Here, 1 indicates that $y_{i}$ is the same as and belongs to the same class. The pseudo-code for the AWRN training algorithm process is shown in Algorithm 1.

**Algorithm 1: Training algorithm for the AWRN (n-ways, 
$N_{s}$
-shots).
N is the number of examples in the training set, K is**
**
the number of classes in the training set. n is the number of classes in every episode. RAND(S,N) represents the set of N**
**
classes randomly selected from the set S without substitution. 
$N_{s}$
 is the number of support samples per class. 
$N_{s}$
 is the number of**
**
query samples per class. Episodes denote the number of iterations.**
Input: Training set D = (
$x_{i}$
, 
$y_{i}$
), where i = 1,2…, N, each 
$y_{i}\in \left[1,K\right]$
. 
$D_{k}$
 is the subset of D, containing all elements that satisfy 
$y_{i}$
 = k.Output: The optimal model 
$F_{\theta }$
, 
$A_{\theta }$
, 
$S_{\theta }$
 for the classification of test datasets.1. Model initialization: Feature extraction module 
$F_{\theta }$
, parallel attention-weighted module 
$A_{\theta }$
, similarity contrast module 
$S_{\theta }$
2. for i = 1 to episodes do3. 
$V\leftarrow Rand\left\{\left(1,2,\cdots ,K\right),n\right\}\ldots$
 Select class indices for episodes4. for k in 
$\left\{1\ldots n\right\}$
 do
$S_{k}\leftarrow Rand\left\{D_{v_{k}},N_{s}\right\}▽$
 Select support samples
$Q_{k}\leftarrow Rand\left\{D_{v_{k}}\setminus S_{k},N_{q}\right\}▽$
 Select query samples5. Forward update 
$L_{MSE}\left(\theta_{F_{\theta }},\theta_{A_{\theta }},\theta_{S_{\theta }}\right)▽$
 update loss6. Backward update 
$F_{\theta }$
, 
$A_{\theta }$
, and 
$S_{\theta }$
 ▽ update model7. end for8. end for

The parameters of the model, assuming $\theta_{F_{\theta }},\theta_{A_{\theta }}$ and $\theta_{S_{\theta }}$ parameters of the feature extractor module $F_{\theta }$ , the attention-weighted module $A_{\theta }$ , and the similarity contrast module $S_{\theta }$ , respectively, are updated according to the results of the loss function. The objective formula for parameter training is as follows:

(13) $$\theta_{F_{\theta }}^{*},\theta_{A_{\theta }}^{*},\theta_{S_{\theta }}^{*}\leftarrow \underset{\theta_{F_{\theta }},\theta_{A_{\theta }},\theta_{S_{\theta }}}{argmin}L_{MSE}\left(\theta_{F_{\theta }},\theta_{A_{\theta }},\theta_{S_{\theta }}\right)$$

The optimal parameters $\theta_{F_{\theta }}^{*}$ , $\theta_{A_{\theta }}^{*}$ and $\theta_{S_{\theta }}^{*}$ can be obtained by solving the objective function shown in (13).

### 3.3. AWRN-Based Valve Fault Diagnosis Method

Information theory plays a crucial role in troubleshooting valve energy conversion systems. The analysis of displacements and gas chamber pressures within valve energy conversion systems is employed to identify and pinpoint faults.

**Figure 4:**
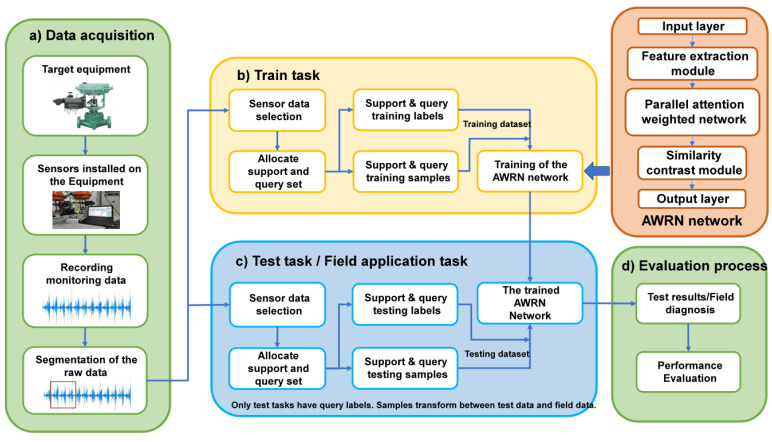
Flow chart of the AWRN-based valve fault diagnosis method.

The process of the AWRN-based valve fault diagnosis method is presented in Figure 4. Firstly, data are collected from the sensors set up for the target valve and processed by segmentation. Secondly, the processed data are fed into the AWRN network for training, resulting in a final trained AWRN network. Thirdly, the network performance is tested through test tasks and field application tasks. Finally, the network performance is evaluated by comprehensively analyzing the results obtained from testing and field diagnosis. The specific program for each step is as follows:

(a) Data acquisition: The target is selected, and a program is developed for configuring sensors to the target valve. The acquisition device records the sensor data, segmented according to 1600 sampling points to generate the original data set.
(b) Training task: To begin, the training set is selected for the model training task. Next, the training set is divided into support sets and query sets. The support and query sets are then trained in the AWRN network. The training process consists of three steps:
  1. The feature extraction module generates 3D features (category–channel–sample).
  2. The parallel attention-weighted module is used to obtain new weighted features.
  3. The similarity contrast module is used to calculate similarity scores, resulting in a well-trained AWRN network.
(c) Test task/field application task: The test task and field application task are similar, except that only the test task has a query set label. The sample data in the process switches between test data and field data as the task changes. Firstly, the testing set as well as the field data set is selected for the model test task or the field application task. Secondly, the test task divides the testing set into a support set and a query set, while the field application task divides the field data set into a support set with labels and a query set without labels. Thirdly, the test task/field application task feeds the support and query sets into the AWRN network for testing.
(d) Performance evaluation: The test task compares the results obtained from the test with the label results and outputs the test results and accuracy for performance evaluation based on classification performance. The field application task outputs test results directly for performance evaluation.

## 4. Dataset and Experimental Setup

The primary objective of fault diagnosis methodology is to examine the system, using either frequency or time-domain analysis, to pinpoint the root cause of a fault when it occurs. Initially, we analyze the time-domain signals generated by the DAMATICS model to simulate and create a typical fault dataset within a valve energy conversion system. Additionally, we provide the PU dataset containing frequency signals to assess the network’s reliability.

### 4.1. The Construction of DA Valve Dataset

#### 4.1.1. Basic Introduction and Model Building

**Figure 5:**
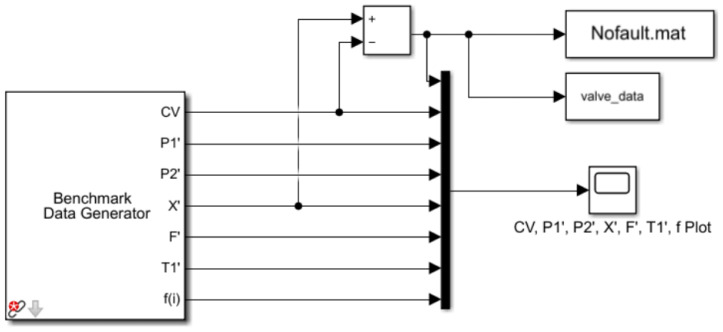
Data generation model.

DAMATICS [3] is a troubleshooting benchmark that consists of a process simulator and real data from electro-pneumatic actuators used in a Polish sugar plant. The benchmark includes a total of 19 fault tests in the four main functional blocks, such as positioner faults, servo motor faults, controller faults, and general/external faults. However, the small volume of real data, containing only four general/external faults, is not suitable for network training [38]. Therefore, we utilized the DAMATICS process simulator to generate the fault data. Based on this model, we have improved the way the data are acquired, and the structure of the model is shown in Figure 5.

The benchmark data generator generates timing signals, which are then processed and stored in a mat file. To ensure the comprehensiveness and technical nature of the experiment, we specifically selected six faults that are more typical and difficult to distinguish, as well as one health state for analysis. The selected faults are listed in Table 3.

**Figure 6:**
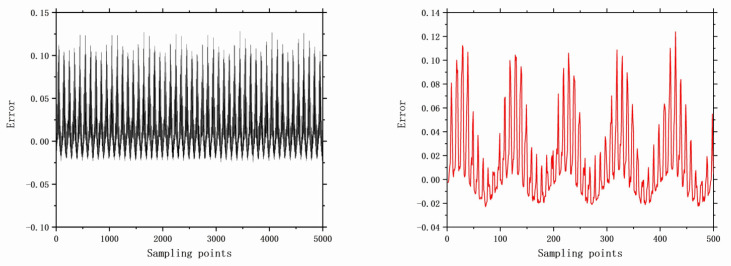
No faults with 5000 samples and 500 samples.

**Figure 7:**
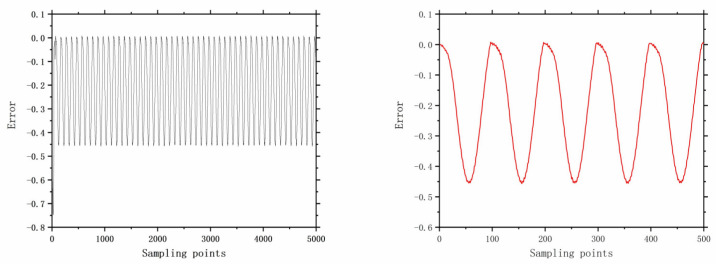
The error of F7 with 5000 samples and 500 samples.

**Figure 8:**
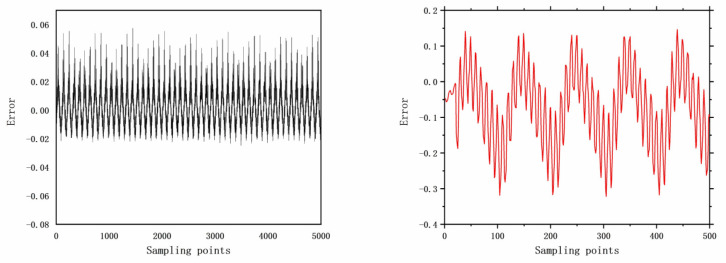
The error of F10 with 5000 samples and 500 samples.

**Figure 9:**
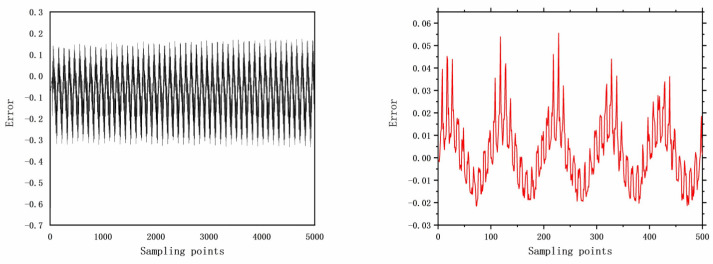
The error of F12 with 5000 samples and 500 samples.

**Figure 10:**
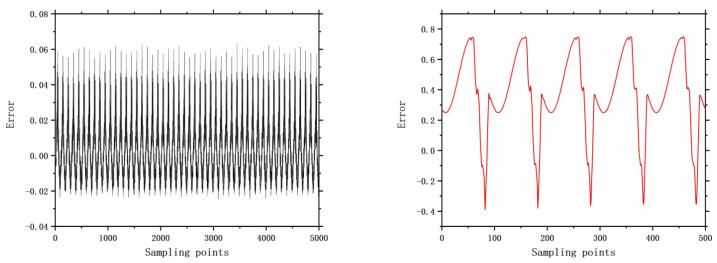
The error of F15 with 5000 samples and 500 samples.

**Figure 11:**
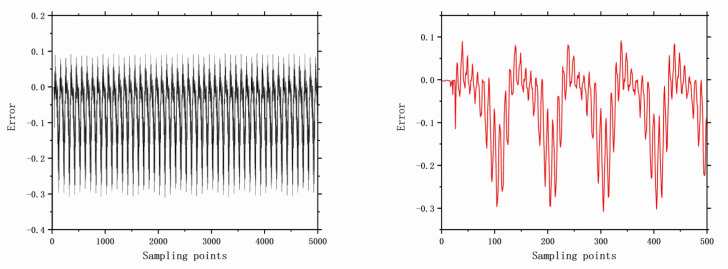
The error of F16 with 5000 samples and 500 samples.

**Figure 12:**
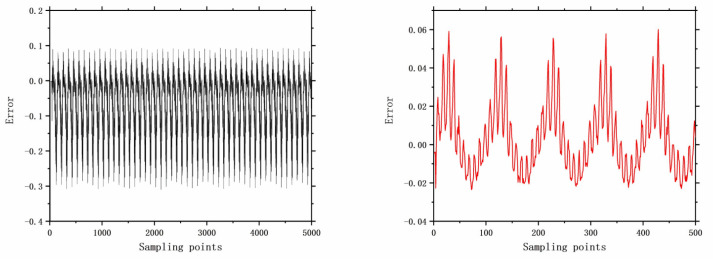
The error of F17 with 5000 samples and 500 samples.

The faults selected for analysis include control valve faults, pneumatic servo motor faults, positioner faults, and general external faults. The model parameters are determined based on the type of fault, and the error data are generated using a data generation model. The datasets are acquired through multiple acquisition points in succession, and the time-domain signals for these health and faults are illustrated in Figure 6, Figure 7, Figure 8, Figure 9, Figure 10, Figure 11 and Figure 12.

#### 4.1.2. The Dataset Construction Steps

(a) Model initialization: First, calculate the relative error value by subtracting the displacement output value from the given signal value. This relative error value represents the variation in displacement signals across different cases. Save this value to a mat file. Additionally, set the basic parameters, such as the simulation time (256,000), fault start time (0), and end time (inf).
(b) Data acquisition: Obtain simulation data under different conditions by varying the fault type, fault degree, and adding noise options. Specifically, we generate simulation data for seven different conditions, including both noisy and noise-free cases, with a large fault degree.
(c) Data processing: Remove the first 200 data points from the simulation data to obtain stable fault conditions. The simulation data are represented as a two-dimensional matrix, where the first row represents the sampling points and the second row represents the displacement error values.

### 4.2. The Introduction of PU Dataset

The aim of using the PU dataset [39] in our study is to assess the efficacy of the network introduced in this paper. Demonstrating its validation on a publicly accessible dataset serves as a robust means to establish the network’s reliability. Experiments use the PU dataset conducted by Paderborn University. This dataset consists of three types of faults affecting gears: outer ring faults, inner ring faults, and inner and outer ring faults. Different operating conditions in the dataset produce vibration signals with unique characteristics that are used to indicate the health of the gears. The dataset includes both artificially induced and naturally occurring damage, with piezoelectric accelerometers used to collect vibration signals from bearing housings, sampled at 64 kHz. The dataset comprises vibration signals generated by gears under various operating conditions, with 300 samples for each state, and each sample is a time series of length 1024. The dataset also includes labels for each sample indicating its operating state.

To assess the generalization and reliability of the selected network, this paper selected 14 representative categories from the PU dataset, including 1 healthy bearing, 4 naturally faulty bearings, and 8 artificially damaged bearings, for the classification task under operating conditions N09_M07_F10. The working conditions are shown in Table 4.

### 4.3. Experimental Setup

To ensure a fair comparison, we replicated a portion of the network using the PyTorch framework and compared our results with those reported in typical published papers. Our experiments were conducted on a computer with an 11th Gen Intel(R) Core (TM) i7-1165G7 processor @ 2.80 GHz, 16 GB RAM (Intel, Santa Clara, CA, USA), and 64-bit Windows 11 OS.

Initially, we adjusted the hyperparameters of both networks and found that a reduction ratio of 8 and a convolution kernel size of 3 × 3 produced better results after several experiments. Table 5 shows the setting of the model hyperparameters, including the number of channels, reduction ratio, number of categories in the sample, the number of support samples per category, batch size, and number of iterations. We trained the model using the Adam optimizer with a learning rate of 0.001 and reduced the learning rate to half for every 1000 epochs trained. Cross-validation was used to evaluate the classification properties.

## 5. Experimental Results

We evaluated the performance of the AWRN on two datasets: the DA valve failure dataset and the PU gear failure dataset. We compared the AWRN network with several typical networks and found that it significantly improved the expression of the network, demonstrating its effectiveness. In addition, we conducted attention comparison and ablation experiments to determine the optimal parameters for the attention models. Our experimental results validate the broad applicability of this architecture to different attention models and tasks. Finally, we discussed the effects of reduction ratio, expanded convolution kernel size, and sample size on model accuracy. It has been observed that the selection of an appropriate attention framework along with specific hyperparameters can significantly enhance modeling outcomes, thereby improving the classification accuracy of the model.

### 5.1. Comparative Experiments Using Different Models

Due to the limited size of the public DA dataset, which only contains four types of faults, it is difficult for the fault data to satisfy both the training and testing requirements of the network. Therefore, many current papers use its process simulator to generate the fault data [40,41,42,43]. However, the selection of fault samples and different sampling methods may lead to unfairness. To compare the proposed method with commonly used methods for valve fault diagnosis, this paper uses the three-way and six-shot approach as an experimental comparator. Specifically, the following methods are compared:

(1) UAE, an unsupervised deep neural network that reconstructs the input through a compressed latent representation using encoders and decoders, with a separate encoder and decoder for each channel.
(2) TCN, a time convolution network that stacks TCN residual blocks for the encoder and replaces the convolution in the TCN residual blocks with transposed convolution for the decoder.
(3) LSTM, a long short-term memory recurrent neural network that models the process of data generation from potential space to observation space and is trained using variational techniques.

The model comprising PCA, UAE, LSTM, and TCN comes from Reference [9]. As shown in Table 6, the AWRN network was compared with PCA, UAE, LSTM, and TCN networks on DA after extracting fault features and obtaining classification results through experiments. The proposed AWRN network achieves the highest classification accuracy among the above algorithms. Compared with the best-performing UAE, the AWRN network achieved 2.68% higher classification accuracy on DA. The comparison results validate the effectiveness of the AWRN network and show that the AWRN network can effectively solve the problem of the problem of accurately diagnosing pneumatic valve faults with limited sample data.

Using the five-way approach as an experimental comparator in the PU public dataset, the proposed method is compared with the commonly used fault classification methods in few-shot learning to comprehensively evaluate the network performance.

(1) RRN: Strengthening the relation network, replacing the feature extractor in RN with a transfer learning model, and using sticky note smoothing and the Adm optimizer to improve the classification accuracy.
(2) MAML: Using several different tasks to train the model, and using the training data from these tasks to the inner and outer loop of the initial parameters so that it can quickly adapt to new tasks.

As shown in Table 7, after extracting the fault features and obtaining the classification results experimentally, the AWRN network was compared with CNN, RRN, Siamese Net, MAML, and Prototypical Networks on DA. The AWRN network achieved the highest classification accuracy among these algorithms. On PU, the AWRN network achieved an average classification accuracy of 1.09% higher than RRN. Moreover, with different sample sizes, the AWRN network outperformed the other algorithms, achieving a maximum classification accuracy of 98.75% in five-shot.

**Figure 13:**
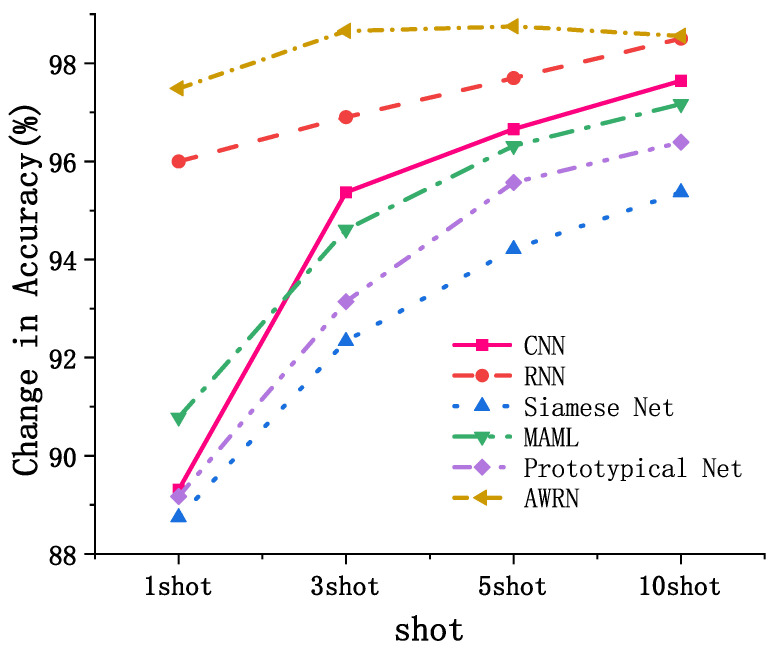
Comparison of trends in different approaches.

As shown in Figure 13, the accuracy of conventional methods rises with the continuous increase in the number of data samples. In contrast, AWRN is specifically suited for scenarios with fewer samples, exhibiting overfitting in cases with an excess of samples, thereby diminishing accuracy. Overcoming this challenge constitutes a primary concern for AWRN. Nevertheless, in the context of this study, AWRN’s accuracy surpasses that of other networks by a significant margin. These results demonstrate the effectiveness and generalization ability of the AWRN network, with superior classification accuracy for various fault diagnosis tasks.

### 5.2. Comparison of Attention-Weighted Methods

**Figure 14:**
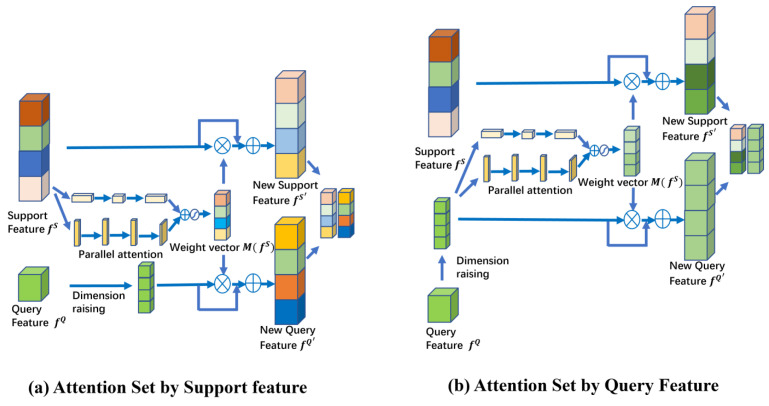
Comparison of two attention-weighted methods.

In the attention-weighted method, two approaches are employed for weight generation. The first method generates weight vectors from the support set features to be applied to both support set and query set features. The second method generates weight vectors exclusively from the query set features. A visual representation of these two distinct attentional weighting methods is provided in Figure 14.

The average accuracy for both the DA dataset and the PU dataset is computed through validation on both datasets, and the results are presented in Table 8 and Table 9.

Based on our findings, we can draw the conclusion that the accuracy results achieved through attention weighting using support set features significantly outperform those obtained when employing attention-weighted with query set features. The figure also illustrates the rationale behind this observation. When using query set features for attention weighting, although it enhances the similarity among similar features, it simultaneously introduces ambiguous features among dissimilar features. It is this ambiguous feature that leads to a substantial decline in overall classification performance and can even result in network instability. In conclusion, under typical circumstances, attention mechanisms prove advantageous in enhancing the performance of fault classification, but this is not a universal certainty. The introduction of attention mechanisms should be carefully selected based on the characteristics of the methodology.

### 5.3. Attention Ablation Experiments

To evaluate the generalization of the proposed architecture, we conducted experiments by fusing different attention networks such as channel attention (SE), BAM, and CBAM, while making some improvements to these networks. In these experiments, we set the reduction ratio, r, to 8 and the convolution kernel size to 3 × 3, and measured the accuracy of the DA dataset. The results are presented in Table 10 and Table 11.

This ablation experiment involved removing the attention-weighted module to obtain experimental results for the individual relation networks.

The proposed AWRN, using a parallel attention-weighted module, achieved the highest classification accuracy for both one-shot and five-shot in the aforementioned algorithm. Analysis of the experimental results led to the following conclusions:

1. Fusing various attention mechanisms such as channel attention, serial attention, and parallel attention on the AWRN network resulted in higher accuracy than the traditional relation network. The weighted structure of AWRN can be fused with multiple attention networks, making it highly applicable to different attention models.
2. Networks that incorporate both channel attention and spatial attention mechanisms have higher average accuracy than those that use only the channel attention mechanism. This is because the fault data, after passing through the feature network, become three-dimensional vectors with spatial correlations. Thus, incorporating both types of attention mechanisms increases the reliability of the weight vector.
3. The AWRN network with a parallel attention-weighted structure achieved the highest classification accuracy. This is because the parallel structure of attention is more adaptable to the network structure of AWRN, resulting in more representative weighting parameters compared to the serial structure.

**Figure 15:**
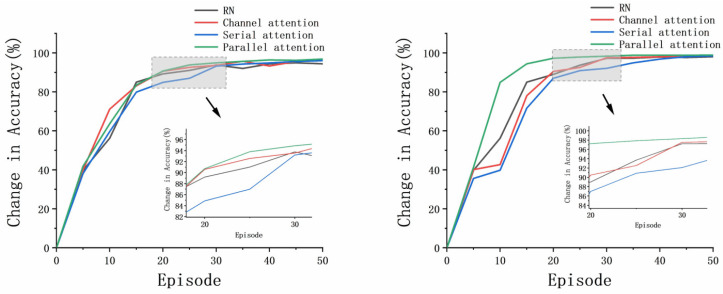
The accuracies of different tasks and sample sizes: one-shot and five-shot of DA.

**Figure 16:**
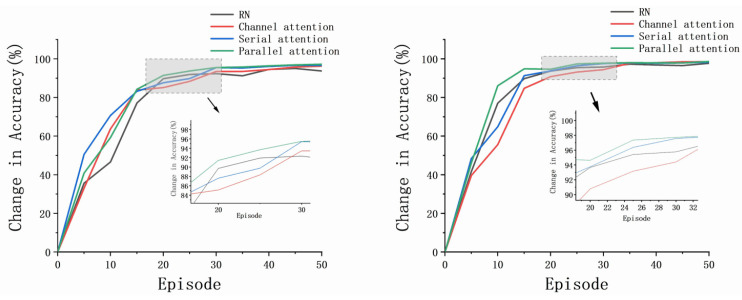
The accuracies of different tasks and sample sizes: one-shot and five-shot of PU.

When evaluating a model, an essential factor to consider is its ability to adapt quickly to the task. Fast adaptation allows for a reduction in the number of required training sessions and computation for the model. It has been observed that as the number of episodes increases, the accuracy rate gradually stabilizes at a fixed value between 20 to 30 episodes. Therefore, to assess the model’s fast adaptation, we compare the accuracy rate during the episodes. Figure 15 and Figure 16 demonstrate how the accuracy changes for the four models with increasing episodes.

The results indicate that the AWRN incorporating the parallel attention mechanism achieves the highest accuracy in both one-shot and five-shot conditions for both datasets. This suggests that the network can quickly adapt to different tasks and sample sizes, resulting in improved classification accuracy.

### 5.4. Experiments on the Selection of Reduction Ratio Parameters

**Figure 17:**
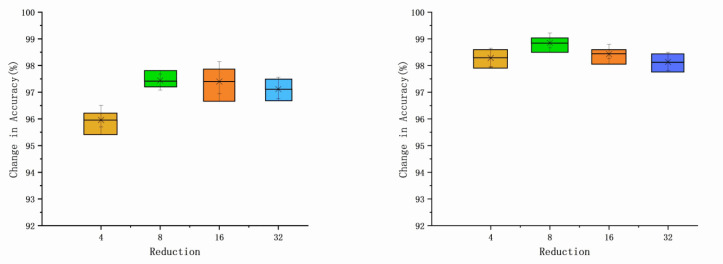
Relationship between reduction ratio and accuracy: one-shot and five-shot of DA.

**Figure 18:**
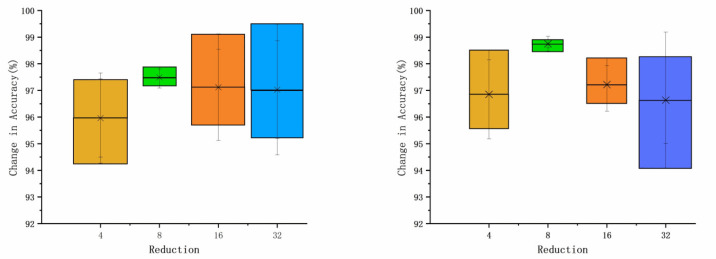
Relationship between reduction ratio and accuracy: one-shot and five-shot of PU.

The results demonstrate that the reduction ratio is directly related to the number of channels in the channel attention and spatial attention branches. When applying AWRN to extract weight vectors, a key point is the choice of the reduction ratio r in the model. choosing a certain decay rate can better control the capacity and overhead of the module and affect the ability of the model to extract weight vectors. the value of r is taken as 4, 8, 16, 32, and the average accuracy of the DA dataset and PU dataset is calculated and the results are shown in Figure 17 and Figure 18.

It can be seen that as the reduction ratio, r, increases, the accuracy rises and then falls, reaching a maximum when r is 8. This indicates that as the reduction ratio increases from 4 to 8, the accuracy increases, although the capacity decreases. As r continues to increase, the accuracy decreases slightly. This may be because the capacity is too small, causing the network to ignore some weighting features during the reduction process, which leads to a decrease in accuracy.

### 5.5. Experiments on Dilated Convolution Kernel Size Selection

**Figure 19:**
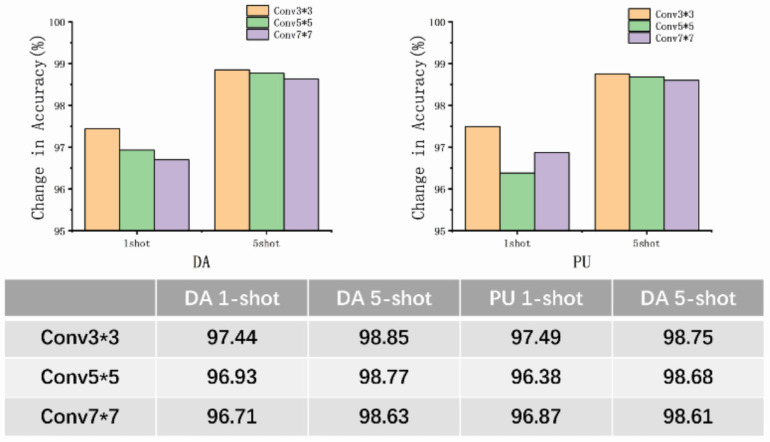
Relationship between convolution kernel size and accuracy.

The use of a certain dilated convolution kernel in spatial attention increases the perceptual field size, which is important for aggregating contextual information in spatial branches. When applying AWRN to a classification task, the selection of the size of the dilated convolution kernel directly affects the representativeness of the new features. The selection of a certain dilated convolution kernel affects the quality of the weighting parameters and allows for a more reasonable distribution of the weighting parameters. The convolution kernels were taken as 3 × 3, 5 × 5, and 7 × 7, respectively, and the average accuracy of the DA dataset and PU dataset was calculated, and the results are shown in Figure 19.

As can be seen from the figure, the classification accuracy is slightly higher when choosing a smaller 3 × 3 convolution kernel. When targeting simple 1D signals like the time domain, choosing too large a perceptual field can result in a lot of spurious features, which occurs for both one-shot and five-shot. Therefore, this situation is improved when a smaller dilated convolution kernel is chosen, and the overall classification accuracy is improved.

### 5.6. Relationship between Sample Size and Precision

**Figure 20:**
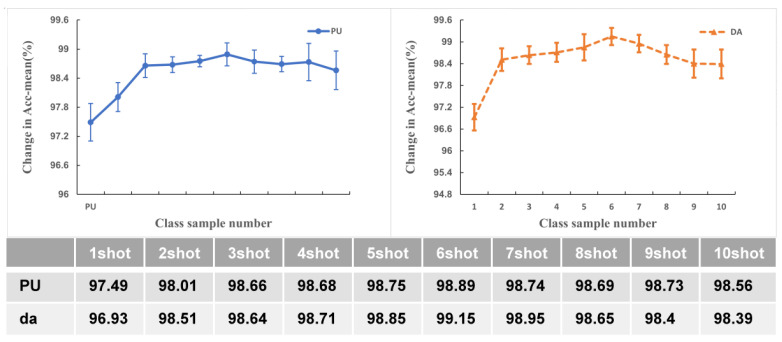
Relationship between the sample size and precision.

A large body of literature demonstrates that as the sample size increases, a few-shot network can learn more features, and that the number of samples determines the network’s thickness. When applying AWRN to a classification task, the selection of sample size is crucial, and selecting a certain sample size will increase the thickness of the network and affect the quality of the weight parameters in the model. The value of ‘shot’ is varied from 1 to 10, where the step size is set to 1, and the average accuracy of both the DA and PU datasets is calculated, and the results are shown in Figure 20.

It can be seen that as the number of shots increases, the accuracy rises and then falls, and reaches a maximum when the shot is six. Meanwhile, in the process of rising, the accuracy rises faster from one-shot to three-shot, while three-shot to six-shot rises slower. After the six-shot, the accuracy falls in the opposite way. This suggests that as the number of shots increases from one-shot to six-shot, the accuracy of the model plummets as the network learns more features at first, and then becomes able to capture fewer new features, making the accuracy rise slower, but the overall accuracy continues to improve. As the number of shots continues to increase, the accuracy drops slightly, probably due to the model parameters being over-tuned and many spurious features being learned into the network, resulting in a drop in accuracy. If given a higher number of shots, it would cause the accuracy of the model to plummet, or even cause the model training to crash.

## 6. Results

As an energy conversion device, a pneumatic control valve may experience unpredictable operational abnormalities potentially diminishing the system’s reliability. Generally deep learning requires many samples, but faults such as pneumatic valves often exist where it is difficult to obtain a large number of samples to support high-performance fault diagnosis. For the problem of accurately diagnosing pneumatic valve faults with limited sample data, this paper proposes a few-shot valve fault detection method based on a weighted attention relationship network (AWRN) to improve feature extraction capability and network classification accuracy for efficient detection of valve faults. In order to verify the effectiveness of the method, a DA valve fault dataset is constructed, and experimental validation is performed on this dataset and another benchmark PU gear fault dataset. The experimental results show that the proposed AWRN network, with an accuracy of 99.15% on DA and an average accuracy of 98.37% on PU, compared with typical fault diagnosis methods, the performance of the proposed method is superior, and the method can still guarantee a high accuracy rate with a significantly lower amount of data for training. More importantly, the AWRN network proposed in this paper has strong generalization capability and wide applicability to different attention models and different tasks. The work in this paper provides a new approach to achieve valve fault detection and classification with small sample data. In our future work, the proposed method will be applied to various energy conversion systems, encompassing components such as gears and control valves, to facilitate offline diagnostics and analyze failures. Simultaneously, we plan to optimize the network structure to enhance its resilience against overfitting.

## Figures and Tables

**Table 1:** Basic operation symbols in few-shot learning.

Notation	Meaning
$x_{i}^{S}$	Input support set
$x_{j}^{Q}$	Input query set
*i*	Each of these categories, ranging from 1 to k.
*j*	The number of simultaneous branches per iteration
*k*	The number of categories
$a\in R^{k\times H\times W}$	Vector
$f^{S}$	Support set feature
$f^{Q}$	Query set feature
$f^{S^{\prime }}$	New support set feature
$f^{Q^{\prime }}$	New query set feature
⊗	Kronecker product
$L_{MSE}$	Mean squared error
$r_{i,j}$	Similarity score
D	Concatenation of feature maps at depth
AWRN	Attention-weighted relation network

**Table 2:** Some definitions in relation networks.

	Description
Support set	The support set is made up of a small number of data sets, acting as a reference for the query set
Query set	The query set is made up of a small number of data sets to train the network parameters by the degree of matching between the query set and the support set
Source domain	The source domain is a dataset that has been acquired prior to the learning task and used for training and modeling. Source domain data are typically collected from one or multiple related domains and often come with labels or manual annotations
Target domain	The target domain can be newly collected data outside of the source domain or a subset of samples within the source domain that have not been seen before
Training set	A collection of data from the source domain to train a few-shot learning model, usually consisting of a support set and a query set
Testing set	Data from target domain sets for evaluating the performance of few-shot learning models
shot	Number of samples per category used to train the model
ways	Number of categories in a few-shot learning task

**Table 3:** Typical Valve Failures.

Serial Number	Fault Description	Fault Category
F7	Media evaporation or critical flow	Control valve failure
F10	Diaphragm perforation for servo motors	Pneumatic servo motor failure
F12	Electrical converter failure	Positioner failure
F15	Faulty positioner feedback	Positioner failure
F16	Positioner supply pressure drop	General faults/external faults
F17	Sudden changes in pressure outside the valve	General faults/external faults
No-Fault	No-Fault	No-Fault

**Table 4:** Health and damage categories at N09_M07_F10 Working Conditions.

Name	Reasons	Location	Features
KA01	Electric discharge machining	Outer Ring	Artificial damage
KA03	Electric discharge machining	Outer Ring	Artificial damage
KA05	Electric discharge machining	Outer Ring	Artificial damage
KA07	Borehole	Outer Ring	Artificial damage
KA08	Borehole	Outer Ring	Artificial damage
KI01	Electric discharge machining	Inner Ring	Artificial damage
KI03	Manual electric engraving	Inner Ring	Artificial damage
KI05	Manual electric engraving	Inner Ring	Artificial damage
K001	Health	Health	
KA04	Fatigue, electrical erosion	Outer Ring	Natural damage
KB23	Fatigue, electrical erosion	Inner and outer rings	Natural damage
KB27	Plastic deformation, fracture, and cracking	Inner and outer rings	Natural damage
KI04	Fatigue, electrical erosion	Inner Ring	Natural damage

**Table 5:** Setting of model hyperparameters.

Parameters	Size	Description
C	64	Number of channels used to describe the dimensionality of the feature
r	8	Reduction ratio
class	3, 5	Number of categories in the sample
shot	1–10	Number of support samples per category
batch size	10	Number of simultaneous branches per iteration
Episode	100	Number of complete tasks of the agent interacting with the environment

**Table 6:** Accuracy comparison of DA dataset (%).

Model	DA
PCA	82.45
UAE	96.47
LSTM	81.17
TCN	91.77
AWRN	99.15

**Table 7:** Accuracy comparison of the PU dataset (%).

Model			PU		
	**One-Shot**	**Five-Shot**	**Five-Shot**	**Ten-Shot**	**Overall**
CNN	89.31	95.37	96.66	97.64	94.75
RRN [31]	96.0	96.9	97.7	98.5	97.28
Siamese Net [44]	88.75	92.34	94.21	95.37	92.67
MAML [44]	90.78	94.61	96.32	97.17	94.72
Prototypical Net [44]	89.17	93.14	95.57	96.39	93.57
AWRN	97.49	98.66	98.75	98.56	98.37

**Table 8:** Attention ablation experiment for the DA dataset.

Model	DA	
	one-shot	five-shot
AWRN + Query feature	60.85 (±6.74)	61.08 (±16.06)
AWRN + Support feature	97.44 (±0.37)	98.85 (±0.36)

**Table 9:** Attention ablation experiment for PU the dataset.

Model	PU	
	one-shot	five-shot
AWRN + Query feature	39.11 (±4.36)	42.71 (±3.10)
AWRN + Support feature	97.49 (±0.40)	98.75 (±0.28)

**Table 10:** Attention ablation experiment for the DA dataset.

Model	DA	
	one-shot	five-shot
RN	96.84 (±0.24)	98.72 (±0.13)
AWRN + Channel attention	96.98 (±0.55)	98.77 (±0.24)
AWRN + Serial attention	97.34 (±0.32)	98.84 (±0.44)
AWRN + Parallel attention	97.44 (±0.37)	98.85 (±0.36)

**Table 11:** Attention ablation experiment for the PU dataset.

Model	PU	
	one-shot	five-shot
RN	97.08 (±0.33)	98.23 (±0.25)
AWRN + Channel attention	97.45 (±0.51)	98.59 (±0.29)
AWRN + Serial attention	97.21 (±0.65)	98.61 (±0.16)
AWRN + Parallel attention	97.49 (±0.40)	98.75 (±0.28)

## Data Availability

Data are contained within the article.
